# A variable gene family encoding nodule-specific cysteine-rich peptides in pea (*Pisum sativum* L.)

**DOI:** 10.3389/fpls.2022.884726

**Published:** 2022-09-14

**Authors:** Evgeny A. Zorin, Marina S. Kliukova, Alexey M. Afonin, Emma S. Gribchenko, Mikhail L. Gordon, Anton S. Sulima, Aleksandr I. Zhernakov, Olga A. Kulaeva, Daria A. Romanyuk, Pyotr G. Kusakin, Anna V. Tsyganova, Viktor E. Tsyganov, Igor A. Tikhonovich, Vladimir A. Zhukov

**Affiliations:** ^1^All-Russia Research Institute for Agricultural Microbiology, Saint Petersburg, Russia; ^2^Department of Genetics and Biotechnology, Faculty of Biology, Saint Petersburg State University, Saint Petersburg, Russia

**Keywords:** *Pisum sativum* L., nitrogen-fixing symbiosis, root nodules, NCR peptides, transcriptomics, spatiotemporal expression pattern, prediction of antimicrobial properties

## Abstract

Various legume plants form root nodules in which symbiotic bacteria (rhizobia) fix atmospheric nitrogen after differentiation into a symbiotic form named bacteroids. In some legume species, bacteroid differentiation is promoted by defensin-like nodule-specific cysteine-rich (NCR) peptides. NCR peptides have best been studied in the model legume *Medicago truncatula* Gaertn., while in many other legumes relevant information is still fragmentary. Here, we characterize the NCR gene family in pea (*Pisum sativum* L.) using genomic and transcriptomic data. We found 360 genes encoding NCR peptides that are expressed in nodules. The sequences of pea NCR genes and putative peptides are highly variable and differ significantly from NCR sequences of *M. truncatula*. Indeed, only one pair of orthologs (*PsNCR47*–*MtNCR312*) has been identified. The NCR genes in the pea genome are located in clusters, and the expression patterns of NCR genes from one cluster tend to be similar. These data support the idea of independent evolution of NCR genes by duplication and diversification in related legume species. We also described spatiotemporal expression profiles of NCRs and identified specific transcription factor (TF) binding sites in promoters of “early” and “late” NCR genes. Further, we studied the expression of NCR genes in nodules of Fix^–^ mutants and predicted potential regulators of NCR gene expression, one among them being the TF ERN1 involved in the early steps of nodule organogenesis. In general, this study contributes to understanding the functions of NCRs in legume nodules and contributes to understanding the diversity and potential antibiotic properties of pea nodule-specific antimicrobial molecules.

## Introduction

Legumes (family Fabaceae) form a unique group among plants owing to their ability to fix atmospheric nitrogen in symbiosis with nodule bacteria (rhizobia). Most legume plants develop specialized root organs, called root nodules, where rhizobia perform biological nitrogen fixation while hosted within plant cells in special compartments (symbiosomes) (Sprent, 2001; Tsyganova et al., 2018). In legumes belonging to the inverted repeat-lacking clade (IRLC) and Dalbergoids, rhizobia undergo terminal (i.e., irreversible) differentiation into symbiotic forms called bacteroids. This process prompts an increase in cell size, endoreduplication of the genome, and nitrogen-fixing capabilities (Mergaert et al., 2006; Alunni and Gourion, 2016). In other legumes, the differentiation of bacteroids is reversible and their changes from the free-living state are not so pronounced (Sprent et al., 1987; Denison, 2000; Kereszt et al., 2011). It is considered that terminal bacteroid differentiation (TBD) is more beneficial for the macrosymbiont (i.e., the host plant) since it is associated with better nitrogen fixation efficiency and higher plant-to-nodule mass ratio (Oono and Denison, 2010). It should be noted that the process of TBD is gradual, as was shown using a series of pea mutants blocked at various stages of nodule development (Tsyganov et al., 2003).

The TBD is governed by short defensin-like peptide molecules named nodule-specific cysteine-rich (NCR) peptides that are produced in nodule cells and stimulate rhizobia to terminal differentiation (Mergaert et al., 2003; Pan and Wang, 2017). NCR peptides are transported to symbiosomes and (at least several of them) are able to permeate into bacterial cells, thus promoting TBD (Durgo et al., 2015; Durán et al., 2021). The signal peptidase DNF1 guides the NCR peptides to symbiosomes, while a lack in its activity leads to the complete absence of NCRs in symbiosomes and, consequently, to the absence of TBD. This, in turn, results in undifferentiated bacteroids in the *dnf1* mutant (Van de Velde et al., 2010; Wang et al., 2010). The crucial role of NCR peptides for TBD is also supported by the fact that no gene coding for protein/peptide similar to NCRs could be found in nodule EST and genomic sequences of *Glycine max* (L.) Merr. and *Lotus japonicus* (Regel) K. Larsen that form nodules in which the bacteroids have unmodified morphotype (Mergaert et al., 2003; Graham et al., 2004; Downie and Kondorosi, 2021), nor were NCR genes found in the genome of *L. japonicus* (Alunni et al., 2007).

The NCR peptide family is best studied in the genome of the model legume *Medicago truncatula* Gaertn. where more than 700 NCR genes were predicted and over 600 were found expressed in nodules (Mergaert et al., 2003; Montiel et al., 2017). In general, NCR peptides are small (20–50 amino acids long) molecules having highly variable sequences containing four or six cysteines in conserved positions. These potentially form two or three disulfide bridges, whereas other amino acids can vary between different members of this protein family (recently reviewed in Roy et al., 2020). Like evolutionarily related defensins, NCR genes are translated into non-functional pro-peptides from which signal peptides are cut off, resulting in the production of mature NCR peptides. The mechanism by which NCR peptides switch the bacterial lifecycle to the terminal state is still not completely understood; however, it is suggested that this involves the interaction of NCRs with bacterial membranes and intracellular targets (much like the antibiotic effects of defensins) (Mikuláss et al., 2016). However, a detailed analysis of the structure and antimicrobial activity was performed only for some NCR peptides of *M. truncatula*—*MtNCR247, MtNCR335*, and *MtNCR169* (hereinafter Mt and Ps refers to *M. truncatula* and *P. sativum* gene and/or protein, respectively) (Tiricz et al., 2013; Farkas et al., 2014, 2017; Mikuláss et al., 2016; Isozumi et al., 2021). Based on the isoelectric point of the mature peptide, NCRs can be divided into groups of cationic, anionic, and neutral peptides, of which cationic NCRs usually have strong antimicrobial activity *in vitro*, whereas anionic and neutral NCR peptides are soft antibiotics and, at least against rhizobia, do not exhibit high toxicity (Lima et al., 2020; Downie and Kondorosi, 2021). This fact indicates that anionic and neutral NCRs in nodules may not serve to kill bacterial cells, as do cationic NCRs, but perform some other purpose. One hypothesis is that anionic and neutral NCRs may bind to cationic NCRs to attenuate their antibacterial effect (Montiel et al., 2017; Roy et al., 2020). This might explain the interesting and somewhat paradoxical situation where mutations in two NCR-encoding genes (*DNF4* = *MtNCR211* and *DNF7* = *MtNCR169*) lead to preliminary senescence of nodules and death of bacteria inside nodules (Horváth et al., 2015; Kim et al., 2015). NCR peptides are also predicted to be able to bind to proteins and be ranked according to the so-called Boman index that reflects their protein-binding potential [for instance, MtNCR247 with the highest Boman index, 1.7 kcal/mol, can bind to multiple proteins in bacteroids. This is associated with inhibiting transcription, translation, and cell division (Farkas et al., 2014)].

Rhizobia can resist the NCR peptide attack with the help of specific proteins. One of them, BacA in *Sinorhizobium meliloti* or BclA in *Bradyrhizobium* spp., is a membrane transporter critical for symbiosis because it can import NCR peptides into the cytosol, thus removing them from the cell surface (Haag et al., 2013; Guefrachi et al., 2015). Another example is an M16A family zinc metallopeptidase (host range restriction peptidase, hrrP) that can degrade NCRs. This protein contributes to an increase in bacterial proliferation inside the nodules and participates in the control of host–symbiont specificity since its presence can lead to the formation of non-functional nodules without differentiated bacteroids dependent on the *M. truncatula* genotype (Price et al., 2015).

NCR genes are expressed almost exclusively in nodules in successive waves (with maximum expression either in younger or older nodules), which also may be indicative of their different functions and roles in TBD (Maunoury et al., 2010; Nallu et al., 2013; Guefrachi et al., 2014). Also, more detailed studies of gene expression with the use of microdissection showed that the expression level of NCR genes reaches the maximum in different zones of nodules (predominantly, in the interzone where TBD is taking place), but there is still a diversity in expression patterns (Roux et al., 2014). Apparently, specific transcription factors (TFs) control NCR gene expression, and some of them were computationally predicted for *M. truncatula* (Nallu et al., 2013). The detailed information on NCR gene expression patterns is lacking for other legumes.

Although NCRs have a single origin, their evolution has followed different routes in individual legume lineages (Montiel et al., 2017). This is confirmed by the fact that no orthologs of the essential *MtNCR169* of *M. truncatula* were recognized in the genomic and/or transcriptomic data of other legumes, including pea (Horváth et al., 2015). Hence, the study of members of this gene family in different lineages of different legumes should enrich the knowledge of the evolution of plant antimicrobial peptides and their particular features in particular legume species.

Garden pea is an important legume plant, often classified as an orphan crop due to poor knowledge of its genomics and transcriptomics (Smýkal et al., 2012; Pandey et al., 2021). However, the pea has seen considerable progress of late, enabling the characterization of the genes and gene families over the whole-genomic level (Kreplak et al., 2019). Also, a large collection of well-characterized mutants that are defective in nodule development has been made available for the pea, a development facilitating studies of nodule-related genes (Tsyganov and Tsyganova, 2020). The aim of the present study was to describe the NCR gene family in the pea based on a new genome assembly (NCBI accession number: JANEYU000000000) and study its spatiotemporal expression profiles along with other features. Here, we also confirm the fact of clustering the NCR genes in the pea genome and prove that the expression patterns of closely located NCR genes are more similar than those of the remote ones. Finally, we built the co-expression modules that contain sets of NCR genes together with other symbiotic genes and predicated the TFs that may regulate the expression of pea NCR genes.

## Materials and methods

### Plant material, bacterial strain, and growth conditions

Pea (*Pisum sativum* L.) wild-type line SGE (Kosterin and Rozov, 1993) and the corresponding symbiotically ineffective mutant lines SGEFix^–^-1 (*sym40-1*) and SGEFix^–^-2 (*sym33-3*) (Tsyganov et al., 1998) were used.

Seeds were surface-sterilized in concentrated sulfuric acid for 10 min, rinsed in distilled water five times, and germinated on Petri dishes with humidified sterile vermiculite (3 days at 28°C). Five seedlings of each sample were planted into 2-L metal pots filled with sieved and heat-sterilized (200°C, 2 h) quartz sand.

*Rhizobium leguminosarum* bv. *viciae* strain RCAM1026 (Afonin et al., 2017) grown on solid TY medium for 3 days at 28°C was used for inoculation [resuspended in distilled water to a concentration of 10^7^ colony-forming units (CFUs) per liter]. Inoculation was carried out with 250 ml of *Rhizobium* suspension per pot. At the same time, a mineral nutrition solution without ammonium nitrate (250 ml per pot) was added to trigger the symbiotic phenotype under conditions of nitrogen starvation (Sulima et al., 2019). The plants were cultivated in a VB 1014 (Vötsch Industrietechnik, Germany) growth chamber under the following climatic conditions: day/night: 16/8 h, the temperature of 21 ± 1°C, relative humidity of 75%, illumination 600 μmol photons m^–2^ s^–1^. The plants were watered with distilled H_2_O as needed.

### Microscopy

Three-week-old nodules were fixed and processed using the low-temperature embedding procedure as previously described (Tsyganova et al., 2009). For light microscopy, 0.5-μm-thick, resin-embedded sections were cut with a glass knife and collected on slides. Specimens were stained in 5% Toluidine blue in 0.1 mM sodium borate. Sections were examined on a Nikon Eclipse 800 with a Nikon Coolpix 995 digital camera (Nikon Corp., Tokyo, Japan). For transmission electron microscopy, 90–100-nm-thick ultrathin sections were collected on copper grids with 4% pyroxylin and carbon. The grids with sections were counterstained in 2% aqueous uranyl acetate for 1 h followed by lead citrate for 1 min. The sections of nodules were examined and photographed in a JEM-1200 EM (JEOL Corp., Tokyo, Japan) transmission electron microscope at 80 kV.

### Identification of genes encoding nodule-specific cysteine-rich peptides and computational prediction of physicochemical properties of the peptides

The genes encoding NCR peptides were identified in the new assembly of pea cv. Frisson (NCBI accession number: JANEYU000000000; Afonin et al., unpublished) using the searching algorithm Small Peptide Alignment Discovery Application (SPADA) for the discovery of short peptides (Zhou et al., 2013). Peptide sequences shorter than 30 amino acids in length and peptides not containing cysteine were removed by a custom python script.^1^ The sequences of NCR peptides were analyzed using the Antimicrobial Peptide Database with APD3 algorithm: Antimicrobial Peptide Calculator and Predictor (Wang et al., 2016) to predict its physicochemical properties. The IPC 2.0 (Kozlowski, 2021) tool was used to calculate the isoelectric point (pI) for mature NCR peptides (without a signal peptide). A peptide was recognized as cationic at a value of pI above 8.5 and anionic at a value below 6.5; peptides with an intermediate pI value were defined as neutral. The boundary of the signal and mature part of the peptides was predicted using SignalP 6.0 (Teufel et al., 2022).

### Nodulation experiment and sequencing library preparation

At 12, 21, 28, and 42 days postinoculation, the plants of the SGE line were extracted from pots, root systems were rinsed with cold tap water, and the visually pink mature nodules were separated from roots with sterile forceps and snap-frozen in liquid nitrogen. The mutants SGEFix^–^-1 (*sym40-1*) and SGEFix^–^-2 (*sym33-3*) forming white nodules were analyzed at 21 dpi only. Five plants from each pot constituted one biological replicate; three biological replicates were used for subsequent procedures. Total RNA from each replicate was isolated using TRIzol (Thermo Fisher Scientific, Waltham, MA, United States) according to the manufacturer’s instruction, RNA quality was evaluated using gel electrophoresis in 1.5% agarose gel, and the concentration of RNA was measured on a Shimadzu UV mini-1240 spectrophotometer (Shimadzu, Japan). The 3′ MACE sequencing libraries were prepared from RNA samples using a 3′ MACE kit (GenXPro GmbH, Frankfurt am Main, Germany) and sequenced on Illumina HiSeq 2500 at GenXPro GmbH (Frankfurt am Main, Germany). The raw data are deposited in the NCBI SRA database under accession number PRJNA812957.

### Gene expression analysis

For each library, all reads were processed to filter out adaptor sequences and low-quality sequences. Then, all of the clean reads were mapped to the reference *P. sativum* cv. Frisson genome assembly (NCBI accession number: JANEYU000000000) using STAR (ver. 2.7.6a). 1) (Dobin and Gingeras, 2015). In total, from 4 to 13 million clean reads per sample were mapped to the genome. Using the principal component method, it was shown that all samples have a high degree of grouping according to replicates (Supplementary Figure 1).

Differential expression analysis was conducted using DESeq2 (ver. 1.34.0) package (Michael Love, 2017) in R programming environment (ver. 4.1.2). The differentially expressed genes were considered to be significant at the level of the adjusted *p*-value of < 0.05.

The heatmap showing gene expression patterns was based on a 1-Pearson correlation matrix calculated on normalized per million and logarithmic (log2) expression values transformed into a z-score (which gives the number of standard deviations that a value is away from the mean of all the values in the same gene) using edgeR (ver. 3.20.9) (Robinson et al., 2010) and pheatmap function in R. The expression values of the three biological replicates for a particular stage of symbiosis were averaged. All genes with very low expression (less than 10 reads per sample) were discarded.

In order to identify gene expression clusters, Pearson correlation values were calculated. The final dendrogram for analysis by heatmap was built on the basis of the correlation matrix by the complete linkage method.

### Phylogenetic tree construction

All the phylogenetic trees were built using phangorn (ver. 2.4.0) (Schliep, 2011) and ggtree (ver. 1.10.5) (Yu et al., 2017) packages in R on the basis of the alignment of mature peptides of NCR genes obtained by MAFFT program (ver. 37.90) (Katoh, 2002) with G-INS-i option. The maximum likelihood method was used to construct all phylogenetic trees. The phylogenetic trees were evaluated using bootstrap analysis with 1,000 replicates. Each terminal node was colored according to one of the physicochemical properties, namely, the total net charge and the Boman index.

### dN/dS substitution analysis

To evaluate the rates of dS and dnS substitutions, the coding sequences of NCR genes were split into the signal peptide and the mature peptide section. The total set of sequences was divided into clusters according to the percent of identity: each cluster consists of a group of NCR genes with identity from 65 to 95% (all sequences with < 65 and > 95% identity were discarded). The sequences were aligned using ClustalW. The dS and dnS substitution values were calculated in the PAML software package (ver. 4.9j) (Yang, 2007) by the Nei-Gojobori method (Jukes-Cantor correction), which, by counting the number of dN and dnS substitutions, takes into account multiple potential substitutions at the same site. Gaps were removed in pal2nal (Suyama et al., 2006).

### Single nucleotide polymorphism analysis

An analysis of single nucleotide polymorphism (SNP) sites was conducted with the following procedure. First, nodule transcriptome sequencing raw reads of SGE (Zhukov et al., 2015) and cv. Caméor (Alves-Carvalho et al., 2015; Kreplak et al., 2019) pea lines were obtained from NCBI (NCBI SRA accession number: PRJNA267198). Removing low-quality reads and adapter trimming was performed using the BBDuk tool from the BBMap toolkit.^2^ Clean reads were then mapped to the reference genome of cv. Frisson with bowtie2 (ver. 2.3.4.1) (Langmead and Salzberg, 2012). For the analysis of the obtained SNPs, BCFtools (Danecek et al., 2021) and VCFtools (Danecek et al., 2011) were used. SNPs specific to NCR genes were obtained using bedtools (Quinlan and Hall, 2010).

### Localization of nodule-specific cysteine-rich (NCR) genes in the genome and their similarity within and between clusters

Genome-wide localization of NCR genes was visualized in chromoMap (ver. 0.3) (Anand and Rodriguez Lopez, 2022). The percentage of average similarity for the alignment of NCR genes within and between genomic clusters was obtained using the EMBOSS Needle tool (Madeira et al., 2019).

### Construction of co-expression modules, transcription factor prediction, and promoter sequence detection

The co-expression modules of differentially expressed genes were built using CEMiTool (ver. 4.1) (Russo et al., 2018). TFs and their potential targets were identified in co-expression modules using the GENIE3 tool (ver. 4.1) (Huynh-Thu et al., 2010). Potential promoter sequences were searched for in regions with a length of 200 and 1,000 nucleotides at 5′ end of NCR genes by the MEME program (Bailey et al., 2009). The relationships between TF and NCR genes were analyzed and visualized in Cytoscape (ver. 3.9.1) (Shannon et al., 2003).

## Results

### Nodule-specific cysteine-rich (NCR) genes discovery in pea cv. Frisson genome assembly

The search for NCR genes in the pea genome (NCBI accession number: JANEYU000000000) was performed using the SPADA—a specific algorithm for the discovery of short peptides. The known NCR peptides of *M. truncatula* and, in the following iterations, *P. sativum* were loaded in SPADA as a training dataset. A total of 653 sequences were identified after three iterations, and sequences less than 30 aa were filtered out along with sequences that contain less than four or six conservative cysteines and/or lack signal peptides. This left 360 remaining sequences. Of them, 206 were also found in the dataset of Montiel et al. (2017) (sequences with an identity of > 95% at the putative protein level were considered alleles of the same NCR genes), and 154 sequences were novel. For a number of peptides of sufficient length and with four or six cysteines in their sequence, the SignalP algorithms did not predict the cleavage sites of a signal peptide. These peptides were labeled in the dataset as NCR-like peptides (Supplementary Table 1) and were not included in further analysis. As for the nomenclature of the NCR genes and peptides, we kept the names of PsNCR1-PsNCR353 for the sequences from Montiel et al. (2017) and continued numeration for our novel sequences up to PsNCR507.

All 360 NCR genes were found to be expressed in nodules in our experiment (see below); thus, we consider them the core members of the NCR gene family in *P. sativum* cv. Frisson. Similar to *M. truncatula*, NCR peptides encoded by the identified genes of pea can be divided into two groups: group A (97 sequences) and group B (263 sequences), having four and six cysteines in conservative positions, respectively (Supplementary Table 1).

**FIGURE 1:**
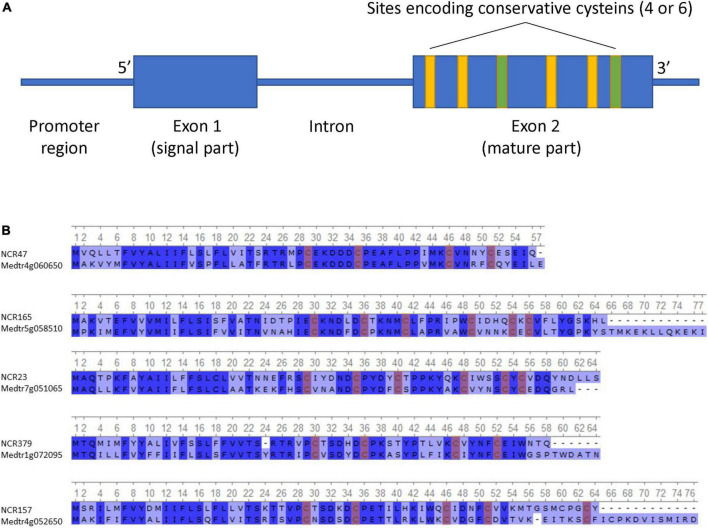
**(A)** Generalized scheme of NCR gene structure. **(B)** Alignment of the top 5 most identical NCR peptides in *P. sativum* and *M. truncatula* (conservative sites are highlighted in blue, and conservative cysteines are highlighted in red).

Most of the identified NCR genes, such as that of *M. truncatula*, were composed of two exons separated by an intron (Figure 1A). The first exon, in most cases, encodes the signal peptide, and the second encodes the mature peptide. The length of the first exon varies from 45 to 138 bp, the second exon from 63 to 396 bp, and the intron from 29 to 4,672 bp. Seven percent of NCR genes contain an additional (second) intron with the third exon encoding the last few amino acids of the peptide. The shortest gene was 141 bp, and the longest was 4,880 bp.

The putative NCR peptides deduced from the gene sequences were 46–156 amino acids in length. Some NCRs differ from others by only one amino acid change. In the list of the discovered NCRs, we found peptides translated from previously known genes of *P. sativum* such as *PsENOD3* (Scheres et al., 1990) (*PsNCR34*, according to our naming scheme), *PsENOD14* (Scheres et al., 1990) (*PsNCR66*), and *PsN466* (*PsNCR110*) (Kato et al., 2002).

### Extreme variability of pea nodule-specific cysteine-rich (NCR) genes

The NCR gene sequences from the pea genome were found to be highly variable and greatly different from the corresponding sequences of *M. truncatula*. Pairwise comparison of putative amino acid sequences of NCR peptides showed a low similarity between the sequence sets from *P. sativum* and *M. truncatula* (Table 1 and Figure 1B), as well as within *P. sativum* set (Supplementary Figure 2). Alignment of all sequences of NCR peptides of *P. sativum* against that of *M. truncatula* using a blastp allowed us to establish that the maximal identity on protein level recorded for pea—*M. truncatula* sequence pairs (*PsNCR47*–*MtNCR312*) was 70.9% (Table 1). Only this pair of NCR genes can be considered orthologs since they are located in syntenic regions of *P. sativum* and *M. truncatula* genomes, a fact which is not the case for the other sequences which are the most similar (Table 1). However, no sequences similar to *PsNCR47*–*MtNCR312* were found in genomes of *Cicer arietinum* L., *Trifolium pratense* L., and *Vicia faba* L. Moreover, in the genomic and transcriptomic data of *C. arietinum, T. pratense*, and *V. faba*, we did not find orthologs for any NCR genes of pea. Likewise, no orthologs were found in the pea genome for the well-studied *M. truncatula* NCR genes such as *MtNCR169* (Isozumi et al., 2021) and *MtNCR211* (Kim et al., 2015) (which are indispensable for symbiosis), *MtNFS1* and *MtNFS2* (which encode peptides that eliminate some rhizobial strains such as Rm41 and A145 from nodules of cv. Jemalong) (Wang et al., 2017, 2018; Yang et al., 2017), or *MtNCR335* and *MtNCR247* (encoding peptides with unique physicochemical properties) (Tiricz et al., 2013). This observation confirms that members of the NCR gene family in related legume species underwent independent evolution (Montiel et al., 2017; Downie and Kondorosi, 2021).

**TABLE 1:** List of five *P. sativum* NCRs that are most similar to *M. truncatula* peptides.

*P. sativum* ID	*P. sativum* chromosome location	*M. truncatula* ID	*M. truncatula* chromosome location	% identity	Length alignment	*E*-value score
*PsNCR47*	chr7LG7	Medtr4g060650	chr4	70.909	55	1.35e-27
*PsNCR165*	chr6LG2	Medtr5g058510	chr5	59.375	64	2.9e-27
*PsNCR23*	chr5LG3	Medtr7g051065	chr7	63.793	58	2.49e-26
*PsNCR379*	chr1LG6	Medtr1g072095	chr1	63.158	57	1.22e-25
*PsNCR157*	chr6LG2	Medtr4g052650	chr4	62.264	53	1.8e-23

**FIGURE 2:**
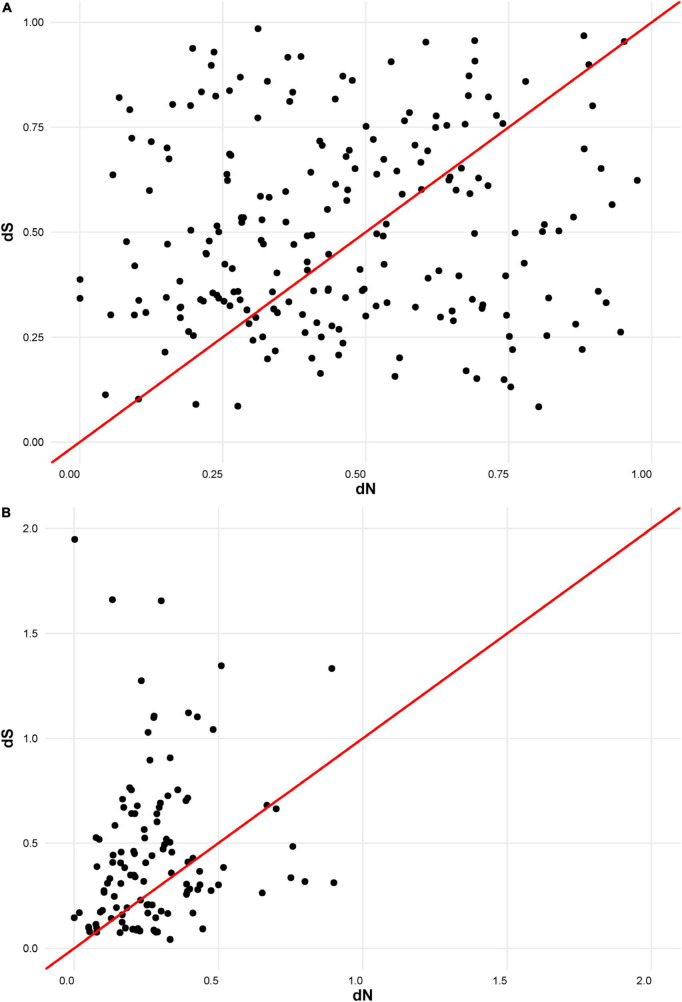
**(A)** dN/dS ratios for mature part of NCR peptides. **(B)** dN/dS ratios for signal part of NCR peptides.

The putative amino acid sequences of the signal peptide were, in general, better conserved than those of the mature peptide, as has been recorded for *M. truncatula* (Alunni et al., 2007). In order to compare the selection pressure on signal and mature peptide parts, we calculated their dN/dS statistics separately. Analysis indicated that the number of synonymous and non-synonymous substitutions is comparable within the mature peptide section. This means that the mature NCR peptides are evolving according to a neutral evolutionary model (Figure 2A). In contrast, within the region encoding the signal peptide, synonymous substitutions prevail against the non-synonymous ones, indicating that this part of NCR genes is undergoing stabilizing selection (Figure 2B).

In order to estimate the allelic polymorphism of NCR genes, cleaned paired-end reads from the pea nodule transcriptome sequencing projects [cv. SGE (NCBI SRA accession number: PRJNA773870) and cv. Caméor (NCBI SRA accession number: PRJNA267198)] were mapped to the genome of cv. Frisson. The single nucleotide variant (SNV) analysis revealed a large number of allelic variants in NCR genes, and NCR genes of SGE line had a greater number of SNVs in the gene coding sequence (Table 2) in comparison to that of cv. Caméor. Thus, NCR genes of the SGE line were more distinct from those of cv. Frisson and cv. Caméor. The pattern of distribution of SNVs by gene region exhibits no significant differences between genotypes (Supplementary Figure 3).

**TABLE 2:** Analysis of SNVs in SGE and Caméor NCR genes as compared to Frisson.

	Total SNV in genes	Total genes with SNVs	No synonymous SNVs	No non-synonymous SNVs
SGE	1,435	247	440	995
Caméor	963	158	251	712

### Physicochemical properties of pea nodule-specific cysteine-rich peptides

The physicochemical properties of pea NCR peptides such as the Boman index and the total net charge were inferred from the putative protein sequences. We noticed that the ratio of cationic, anionic, and neutral peptides in our data differed from that described in Montiel et al. (2017), probably because we used a later-version IPC-2.0 tool built on machine learning algorithms. For adequate comparison, we also recalculated the values of isoelectric points for NCR peptides of *M. truncatula* and *P. sativum* cv. Caméor. Similar to the *M. truncatula* NCRs, anionic peptides prevail among NCR peptides of *P. sativum* cv. Frisson: 126 cationic (34%), 156 anionic (43%), and 83 neutral (23%) (Table 3). The isoelectric pI of pea NCRs ranged from 2.8 to 10.2, and the Boman index varied between −1.09 and 3.88. The distribution of pI and Boman index within the NCR family in all three pea genotypes analyzed was similar to that in *M. truncatula*. Probably due to a large number of amino acid substitutions, the distribution of isoelectric points of mature NCR peptides exhibits slight differences in SGE and Caméor in comparison with Frisson (Table 3).

**TABLE 3:** Distribution of isoelectric points of NCR peptides in *M. truncatula* and *P. sativum.*

	Percent of NCR peptides in *M. truncatula* (cv. A17)	Percent of NCR peptides in *P. sativum* (cv. Frisson)	Percent of NCR peptides in *P. sativum* (cv. SGE)	Percent of NCR peptides in *P. sativum* (cv. Caméor)
Cationic	38%	34%	32%	38%
Anionic	41%	43%	47%	36%
Neutral	21%	23%	21%	26%

**FIGURE 3:**
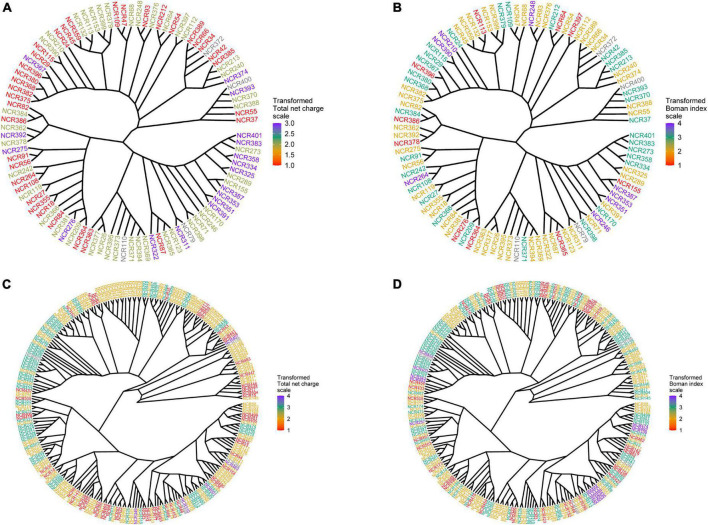
Phylogenetic trees built for groups A and B of NCR genes. Each terminal node was colored according to one of the physicochemical properties. **(A)** Phylogenetic tree for group A colored according to the total net charge values. **(B)** Phylogenetic tree for group A colored according to the Boman index values. **(C)** Phylogenetic tree for group B colored according to the total net charge values. **(D)** Phylogenetic tree for group B colored according to the Boman index values.

The physicochemical parameters of pea NCR peptides were represented on phylogenetic trees constructed separately for peptides of group A (four cysteines) and group B (six cysteines). Each terminal node was colored according to either the total net charge or Boman index (Figure 3). As expected, NCRs with similar physicochemical parameter values were grouped within clades (branches) of phylogenetic trees (i.e., they possibly originate from a relatively recent duplication event); however, many remote clades were characterized with nearly the same physicochemical parameter values (Figure 3), that may indicate convergent evolution of diverse clades of NCR peptides, or may simply be a consequence of the extreme variability of NCR peptide sequences and the high degree of dependence of physicochemical properties on the amino acid composition.

### Localization of pea nodule-specific cysteine-rich (NCR) genes in the genome

**FIGURE 4:**
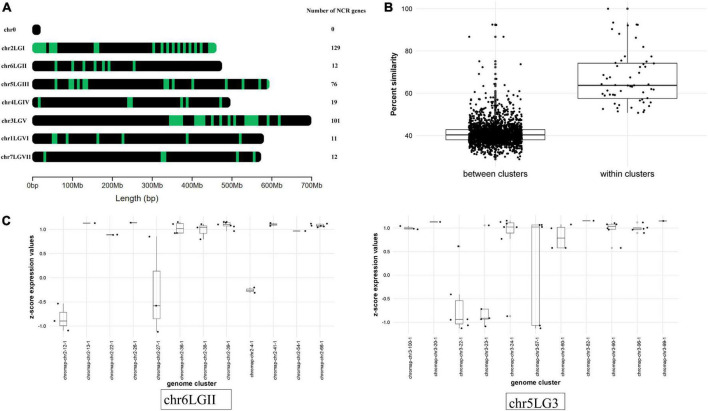
**(A)** Localization of NCR genes in *P. sativum* genome. Green dashes indicate NCR genes. **(B)** Comparison of the average percentage similarity of NCR genes within and between clusters in the genome using boxplots. Each comparison group contains the results of a pairwise alignment of NCR peptides with each other in the form of % similarity of their sequences. The group “within clusters” contains the results of the alignment of peptides among themselves within the same cluster in the genome. The “between clusters” group contains the results of the alignment of peptides belonging to different clusters with each other. **(C)** Evaluation of the NCR gene expression at 12 dpi within and between clusters in the genome. The level of NCR gene expression in clusters is represented by the transformation of log2(CPM) into a *z*-score. Chromosomes with a more pronounced effect are selected for visualization.

Mapping NCR gene sequences to the genome allowed us to reveal a cluster pattern of genomic organization in this gene family in *P. sativum* (Figure 4A). The maximum number of NCR genes (129) is localized on LG1chr2. In order to confirm that the evolution of NCR genes was based on duplication events, we calculated the average percentage of sequence similarity between and within genomic clusters. The boxplots (Figure 4B) clearly demonstrate that the similarity of sequences within clusters on the genome is higher than between clusters. In addition, by analyzing the gene expression data (that is described in detail below) we observed that NCR genes within genomic clusters have a similar level of expression, which supports the hypothesis that a set of genes in a genomic cluster is regulated uniformly (Figure 4C). The expression level in some clusters has a high level of variance, which may be an artifactual result of combining some small clusters of NCR genes into one because of their proximity to each other. Together, these data support the idea that recent duplication events leading to the emerging number of NCR genes played an important role in the evolution of the NCR gene family in pea.

### Expression profiles of the nodule-specific cysteine-rich (NCR) genes

For all identified NCR genes, the analysis of spatiotemporal expression profiles was carried out using data of 3′ MACE sequencing of *P. sativum* wild-type nodules (SGE line) at 12, 21, 28, and 42 dpi and data of RNAseq obtained from microdissected nodules (early zone II, late zone II, and zone III) of the same SGE line at 11 dpi (for a description of methods, see Kusakin et al., 2021).

**FIGURE 5:**
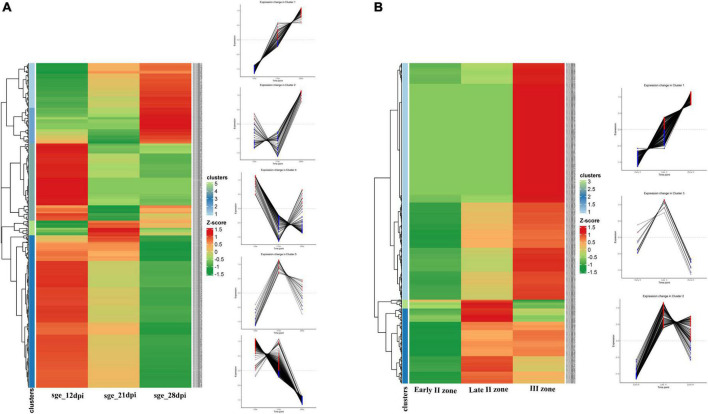
**(A)** Cluster analysis of *P. sativum* NCR genes on the basis of the expression pattern change at the different stages of symbiosis. MACE sequencing data of wild-type line SGE at 12, 21, and 28 dpi. **(B)** Cluster analysis of *P. sativum* NCR genes on the basis of the expression pattern change at the different stages of nodule development. RNA sequencing data of wild-type line SGE in early II nodule zone and late II and III nodule zones. All log2(CPM) expression values were transformed into *z*-score to build heatmaps.

NCR genes were divided into five clusters in accordance with their temporal expression pattern (Figure 5A). The most numerous cluster includes NCR genes, for which the expression level reached its maximum at 12 dpi and gradually decreased to 28 dpi. Large clusters of genes with maximal expression levels at 21 and 28 dpi were also identified. Clusterization data show that the majority of NCR genes are activated prior to 12 dpi. Thus, NCR genes begin to express at various stages of symbiosis. The three main clusters were identified with a maximal expression level at 12, 21, and 28 dpi (corresponding to bacteroid differentiation, nodule maintenance/nitrogen fixation, and initiation of senescence, respectively). Two of them were referred to as “early” and “late” NCR genes with the maximum at 12 and 28 dpi, respectively (Figure 5A). Such a coordinated expression of NCR genes implies that they are regulated by a limited number of TFs.

For analysis of the spatial expression patterns of NCR genes, RNA sequencing data from the nodule microdissection experiment were used (Kusakin et al., 2021). Based on NCR gene expression levels in the early infection zone (zone early II), late infection zone (zone late II), and nitrogen fixation zone (zone III) of the nodule, two main clusters were revealed—a maximum of expression in late II zone and a maximum in zone III (Figure 5B). A small group of NCR genes were also identified whose expression was induced in early II zone, reached a maximum in late II zone, and then was repressed in zone III (Figure 5B).

**FIGURE 6:**
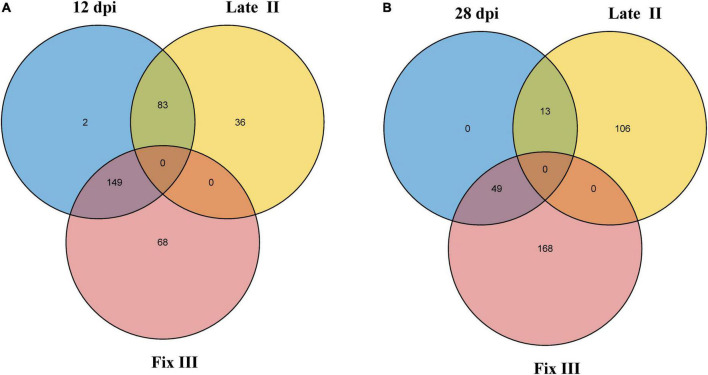
Intersection of NCR genes with expression maximum at different dpi and different nodule zones. **(A)** Intersection of NCRs with maximum expression at 12 dpi and with maximum expression in late II and III zones. **(B)** Intersection of NCRs with maximum expression at 28 dpi and with maximum expression in late II and III zones.

Data from the two experiments match since NCR genes with maximal expression at 12 dpi are expressed in late II and III zones (Figure 6A), while the vast majority of genes with a maximum of expression at 28 dpi are expressed only in nodules’ zone III (Figure 6B). As expected, the “late” NCR genes are active mainly in the nitrogen fixation zone (zone III), while the “early” ones are expressed mostly in the late II zone.

**FIGURE 7:**
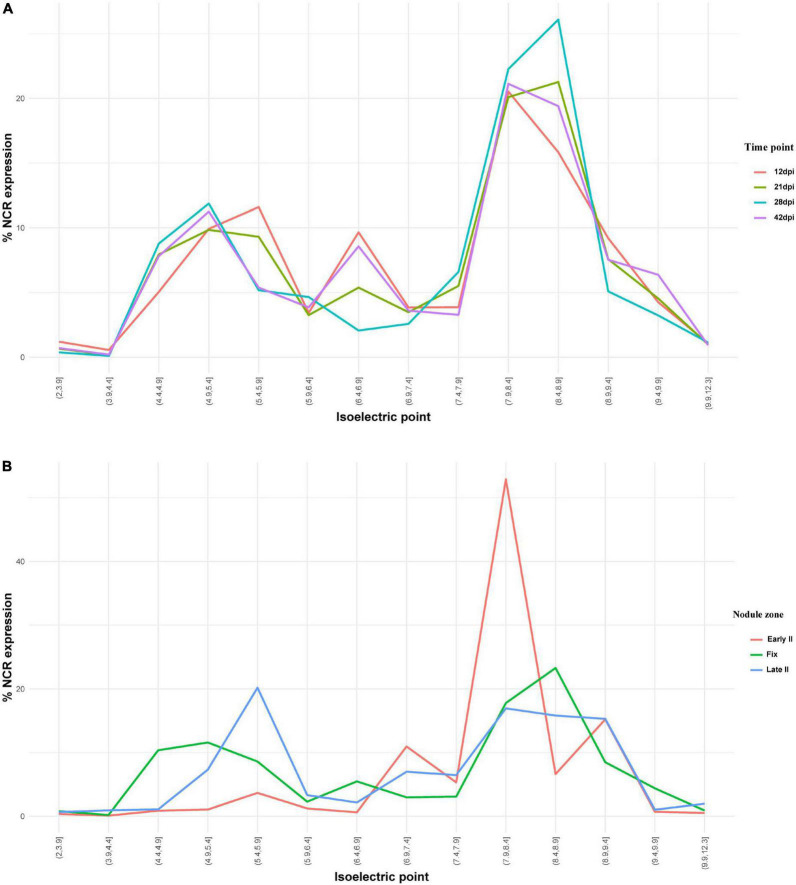
**(A)** Isoelectric point profiles of NCR peptides and the relative expression of NCRs with different Pl across days after inoculation (dpi). **(B)** Isoelectric point profiles of NCR peptides and the relative expression of NCRs with different Pl in different zones of *P. sativum* nodules. % NCR expression is % CPM normalized values of different isoelectric point categories to whole-nodule NCR expression.

NCR genes encoding anionic, neutral, and cationic peptides are expressed relatively uniformly at all studied time points, with maximal number of expressed sequences at 28 dpi (Figure 7A). Cationic peptides were active mainly in the early II zone of the nodule (Figure 7B). Interestingly, two groups of anionic peptides can be distinguished: with pI 4.9–5.4 (the maximal number of which stands at 28 dpi) and with pI 5.4–5.9 (the maximal number of which stands at 12 dpi).

### Expression of nodule-specific cysteine-rich (NCR) genes in nodules of SGEFix^–^-1 (*sym40-1*) and SGEFix^–^-2 (*sym33-3*) mutants

**FIGURE 8:**
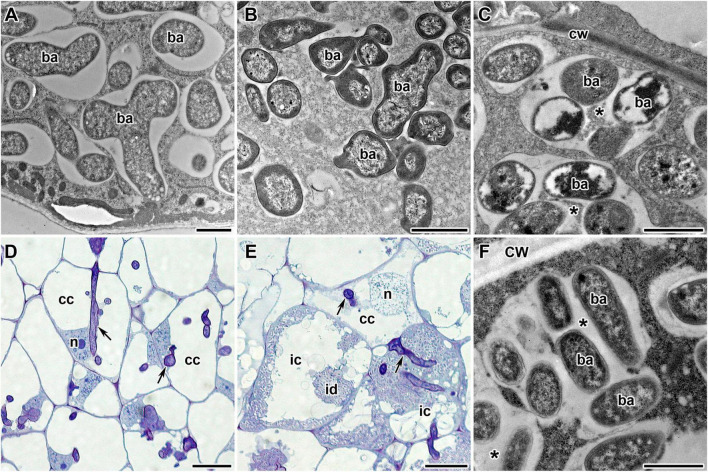
Phenotypes of pea (*Pisum sativum* L.) nodules. **(A)** Wild-type bacteroids. **(B,C)** Bacteroids of mutant SGEFix^–^-1 (*sym40-1*): **(B)** abnormal bacteroids; **(C)** multibacteroid symbiosomes. **(D–F)** Phenotype of nodules of mutant SGEFix^–^-2 (*sym33-3*): **(D)** nodule with “locked” infection threads and no release of bacteria; **(E)** nodule with occasionally released bacteria; **(F)** multibacteroid symbiosomes in the nodule with released bacteria. ic, infected cell; cc, colonized cell; id, infection droplet; n, nucleus; cw, cell wall; ba, bacteroid; *multibacteroid symbiosome. Arrows indicate infection threads. Scale bars = 1 μm **(A–C,F)** and = 20 μm **(D,E)**.

Pea symbiotic mutants SGEFix^–^-1 and SGEFix^–^-2 carry mutations in TF genes *Sym40* = *PsEFD* and *Sym33* = *PsIPD3*, respectively (Tsyganov et al., 1998; Ovchinnikova et al., 2011). Thus, these lines are suitable models for studying the potential link between the activity of TFs EFD and IPD3 and the expression of NCR genes in pea. The mutant SGEFix^–^-1 (*sym40-1*) forms numerous white nodules that, in contrast to wild-type pleomorphic bacteroids (Figure 8A), contain abnormal bacteroids (Figure 8B) and multibacteroid symbiosomes (Figure 8C; Tsyganov et al., 1998). Occasionally, pink nodules with a normal ultrastructural organization are formed. The mutant SGEFix^–^-2 is able to form two types of nodules: white with “locked” infection threads (Figure 8D) and pinkish with rod-shaped bacteroids surrounded by the common symbiosome membrane (Tsyganov et al., 1998). However, the white nodules of some cells form infection droplets prompting the release of bacteria (Figure 8E) that leads to the formation of multibacteroid symbiosomes (Figure 8F; Tsyganov et al., 1998, 2011). In this study, both mutants formed only white nodules.

**FIGURE 9:**
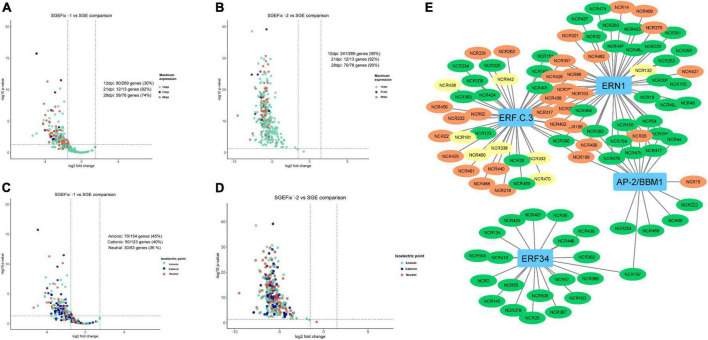
**(A)** Volcano plot showing the distribution of “early” and “late” NCRs among differentially expressed NCR genes in mutant SGEFix^–^-1 (*sym40-1*). **(B)** Volcano plot showing the distribution of “early” and “late” NCRs among differentially expressed NCR genes in mutant SGEFix^–^-2 (*sym33-3*). **(C)** Volcano plot showing the distribution of cationic/anionic/neutral NCR peptides among differentially expressed NCR genes in mutant SGEFix^–^-1 (*sym40-1*). **(D)** Volcano plot showing the distribution of cationic/anionic/neutral NCR peptides among differentially expressed NCR genes in mutant SGEFix^–^-2 (*sym33-3*). **(E)** NCR gene regulatory network based on gene expression data of wild-type and mutants SGEFix^–^-1 and SGEFix^–^-2. NCR genes with a maximum expression at 12 dpi are colored green. NCR genes with a maximum expression at 21 dpi are colored yellow. NCR genes with a maximum expression at 28 and 42 dpi are colored red.

Transcriptomic analysis was performed for mutant and wild-type nodules harvested at 21 dpi. In nodules of SGEFix^–^-2 (*sym33-3*) with no signs of TBD, severe suppression of almost all NCR genes (323 out of 360) was detected, which is in agreement with the phenotype (Figures 9B,D). In nodules of SGEFix^–^-1 (*sym40-1*), in turn, 150 NCRs genes were differentially expressed (downregulated), as compared to SGE nodules. Most of the downregulated NCR genes in SGEFix^–^-1 nodules are assigned to the “late” group (maximal expression at 28 dpi in wild-type nodules), whereas all the genes with no differential expression are from the “early” group (maximal expression at 12 dpi in wild-type nodules) (Figure 9A). At the same time, among the differentially expressed genes in SGEFix^–^-1, the genes encoding cationic, anionic, and neutral peptides are distributed almost equally (Figure 9C).

### Co-expression analysis

**FIGURE 10:**
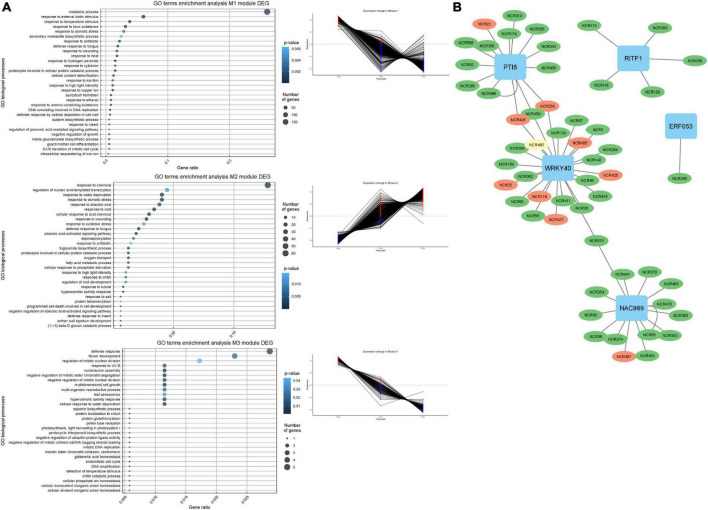
**(A)** Co-expression gene modules and their expression pattern identified using CEMiTool at 12, 21, and 28 dpi and biological process GO enrichment of the differentially expressed genes in detected co-expression modules. **(B)** NCR gene regulatory network based on gene expression data. NCR genes with a maximum expression at 12 dpi are colored green. NCR genes with a maximum expression at 21 dpi are colored yellow. NCR genes with a maximum expression at 28 and 42 dpi are colored red.

In order to get an insight into the potential mechanisms behind the regulation of the NCR gene expression, we conducted a search for gene co-expression modules in MACE sequencing data for time series 12, 21, and 28 dpi. Using CEMiTool, three modules of genes with a high degree of co-expression were detected (Figure 9A). Modules M1 and M3 were enriched with NCR genes with maximal expression at 12 dpi (“early”), while module M2 was enriched with NCR genes with maximal expression at 28 dpi (“late”). The gene ontology analysis showed that modules 1 and 3 were characterized by early activation of biological processes associated with resistance reactions, response to biotic stimuli, ethanol, cytokinins, and response to fungi (Figure 10A). The last two groups may include various symbiotic genes common to both mycorrhizal and nodule symbioses. Module 2 is characterized by overexpression of genes at late stages of symbiosis and is associated with the response to abscisic acid, phosphate starvation, response to chitin, and stimulation of root growth (Figure 10A).

The list of genes co-expressed with NCR genes in these modules was scanned for the presence of TFs genes using the GENIE3 tool. Five TFs potentially regulating the expression of NCR genes were identified: WRKY40, NAC969, RITF1, PTI5, and ERF053 (Figure 10B). Interestingly, NAC969 was found to regulate the expression of “early” NCR genes, while other TFs tended to regulate mainly “late” genes (Figure 9B).

The data of misexpression of NCR genes in the nodules of mutants SGEFix^–^-1 and SGEFix^–^-2 were also subjected to co-expression analysis. With respect to these, the putative TFs that can regulate the expression of NCR genes and thus influence the manifestation of the mutant phenotype in SGEFix^–^-1 and SGEFix^–^-2 were predicted. The list of the TFs identified in our data as potential regulators of NCR gene expression includes ERN1 involved in the early steps of nodule organogenesis and other TFs related to nodule development and functioning, namely ERF.C.3, ERF34, and BBM1 (Figure 9E).

Co-expression modules containing NCR genes were also analyzed for enrichment by biological processes in GO terms. Thus, such biological processes as cellular response to phosphate starvation, response to external biotic stimuli, auxin-activated signaling pathway, reaction to ethanol, and regulation of auxin polar transport are suppressed in the nodules of mutants SGEFix^–^-1 and SGEFix^–^-2. It is worth noting that such biological processes as the defense response to bacteria, the regulation of defense reactions, the response to wounding, and the response to karrikins are increased in mutants compared to the wild type. At the same time, the nodules of SGEFix^–^-1 converge with nodules of SGE by such biological processes as those involved in symbiotic interaction, cellular response to auxin stimuli, cellular response to oxidative stress, starch metabolism, and plant-type hypersensitive response (Supplementary Figure 4).

Additionally, to search for conserved motifs present in promoters of “early” and “late” NCR genes, the 1,000 bp upstream from the translational start site were scanned using the MEME tool. The analysis revealed nine conservative motifs in promoters of NCR genes (Table 4). To identify putative TF binding sites in the promoter regions of NCR genes, we scanned these regions using the SEA program and found different putative TF binding sites for “early” and “late” NCR genes (Table 5). Interestingly, we identified the same conservative motifs in promoter regions of some genes co-expressed with NCR genes [namely, the genes encoding nodulin-13, subunit NF-YB1, gibberellin signaling DELLA protein LA, nodulin-26-like intrinsic protein (NIP), Early nodulin-5, and receptor-like protein CLAVATA2].

**TABLE 4:** (A) The conserved motifs found in the upstream regions of “early” NCR genes using the MEME tool. (B) The conserved motifs found in the upstream regions of “late” NCR genes using the MEME tool.

(A)			
**Motif consensus**	***E*-value**	**Sites**	**Length**
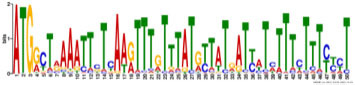	1.2e–302	108	50
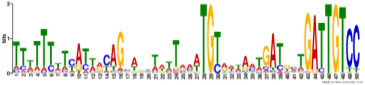	2.0e–179	85	50
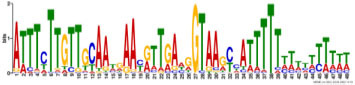	9.4e–105	44	49
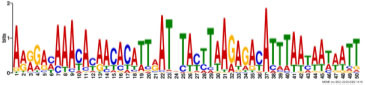	1.4e–102	60	50
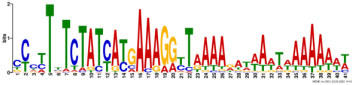	1.2e–070	52	41

**(B)**			
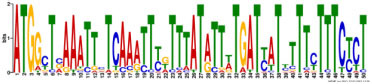	6.2e–140	18	50
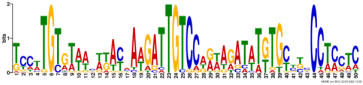	4.6e–088	16	50
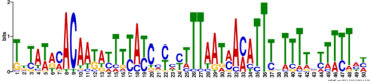	6.5e–053	20	50
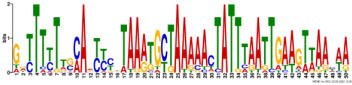	1.1e–050	14	50
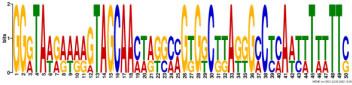	4.5e–039	6	50

**TABLE 5:** (A) Putative TF binding sites in “early” NCR promoter regions predicted by the SEA tool. (B) Putative TF binding sites in “late” NCR promoter regions predicted by the SEA tool.

(A)		
**ID**	**ALT_ID**	**Consensus**
**RWPRK_tnt.NLP7_col_a_m1**	NLP7	WWWTGVCYYTTSRDD
**AP2EREBP_tnt.AT1G71450_col_a_m1**	AT1G71450	CDCCRCCRCCDCCRCCGYCR
**LOBAS2_tnt.AS2_col_a_m1**	AS2	YCDCCGCCGYHDYYKCCGCCG
**BBRBPC_tnt.BPC6_col_a_m1**	BPC6	CTCTCTCTCTCTCTCTCTCTC
**ARF_tnt.ARF2_col_v31_m1**	ARF2	WDSMGACAWR
**C2C2gata_tnt.ZML1_col_a_m1**	ZML1	TCATCATCATCATCA

**(B)**		
**C2H2_tnt.STZ_col_m1**	STZ	CACTNHCACTN
**C2C2gata_tnt.GATA20_colamp_a_m1**	GATA20	TNGATCNGATYND
**Orphan_tnt.BBX31_col_a_m1**	BBX31	AAAAAGTAAAAARDW
**C2H2_tnt.TF3A_col_a_m1**	TF3A	CYTCCTCCTCCTCCTCCTC
**ND_tnt.FRS9_col_a_m1**	FRS9	CTCTCTCTCTCTCTCTCTCTC
**MYB_tnt.MYB55_colamp_a_m1**	MYB55	WGGTWGGTRRRNNDD

## Discussion

Recent success in the development of high-throughput sequencing technologies enables the construction of high-quality genome assemblies for several plant species with large and complex genomes, such as garden pea (Kreplak et al., 2019). These genome assemblies become an invaluable source for deep analysis of gene families encoding small peptides that have usually been overlooked during the analysis of the previous genome and transcriptome assemblies. Indeed, the analysis of Montiel et al. (2017), identified only 353 expressed genes encoding NCR peptides in pea (and 469 in *Medicago sativa* L. and 639 in *M. truncatula*). In our work, the number of detected NCR genes in pea turned out to be essentially the same (360 genes), but among them, we found 154 novel genes. The incomplete intersection of the NCR gene/peptide datasets identified by Montiel et al. (2017) and us can be due to consideration of the allelic variations characteristic for cv. Caméor and cv. Frisson as different genes at our threshold of > 95% protein identity [i.e., many of our novel sequences could be alleles of genes previously described by Montiel et al. (2017)]. Alternatively, the sets of expressed and non-expressed NCR genes could also be different in different pea genotypes and cultivars.

It is also possible that the number of NCR genes in pea and other legume genomes is underestimated [this, however, gives no reason to doubt the conclusion of Montiel et al. (2017) that the degree of TBD correlated with the number of NCR genes]. Moreover, the search algorithms used in our work did not permit us to identify several genes encoding cysteine-rich nodule proteins (namely, *PsN1, PsN6, PsN314*, and *PsN335*) that were previously described for pea cv. Sparkle (Kato et al., 2002). Therefore, the actual number of NCR genes in the genome of cv. Frisson may be even higher. Apparently, analysis of genomes of other pea accessions and cultivars, including the wild ones such as cv. Afghanistan and *Pisum fulvum* Sm. forms, may result in the discovery of other members of the NCR gene family that will lead to a better understanding of its variability and evolution.

The NCR genes in pea are extremely polymorphic, which is true for comparisons of its sequences either within the one genotype or between unrelated genotypes (however, *PsNCR47*, for which the orthologous gene was identified in *M. truncatula*, is not polymorphic at the peptide level in Frisson/Caméor/SGE genotypes). The presence of polymorphism enables estimation of selection pressure using the dN/dS method, and it is clearly seen that the parts of NCR genes undergo different modes of selection pressure [which is also the case for *M. truncatula* nodule-specific NCR and GRP genes (Alunni et al., 2007)]. The parts that encode signal peptides undergo stabilizing (purifying) selection, which is logical given that the peptides must be correctly targeted to specific cell compartments. In turn, the parts encoding mature peptides in pea are evolving neutrally, which, probably, reflects the superposition of some acts of stabilizing selection with respect to some crucial NCR genes and crucial amino acids, i.e., the cysteines, and acts of diversifying selection that leads to an increase in the diversity of NCRs (a factor which is believed to be beneficial for plants). Diversifying (or disruptive) selection is also clearly observed when comparing NCR gene sequences between pea cultivars where the number of non-synonimic changes is much higher than that of the synonymic. In general, this fact, together with the extremely low percentage of similarity between NCR peptides of *P. sativum* and *M. truncatula* (and the absence of orthologous sequences apart for the *PsNCR47*–*MtNCR312* pair) confirms that the evolutionary trajectories of this family are independent in each of the IRLC species. The lack of orthology, which is unusual for symbiotic genes (that are often quite conservative in different legumes), is, however, consistent with the concept that defense proteins of any organism evolve at faster rates than structural or regulatory ones where a minor change in sequence may lead to the serious disturbance of cell function or that of the whole organism.

Like those of *M. truncatula*, pea NCR genes are located in clusters, a fact consistent with the accepted mode of their evolution by duplication and diversification, and they have an uneven distribution between chromosomes: 101 and 129 NCR genes in chromosomes LG5chr3 and LG1chr2, respectively, with fewer on other chromosomes. The NCR gene clusters contain many repetitive sequences and transposons, and many of the surrounding genes in the current pea genome annotation encode short peptides with unknown functions. Apparently, the presence of repeats facilitates unequal recombination events that lead to duplications of NCR genes.

In general, the closer the NCR genes are located to each other the more similar their expression patterns are. This is also a consequence of the recent duplication events that involve promoter sequences along with coding parts of NCR genes. However, diversification and specialization are characteristics not only for coding parts of NCR genes but also for its promoters. The detection of expression waves of NCR genes indicates that some features in promoter regions of different NCR genes have independently evolved for matching different TFs. Indeed, we found different motifs in promoters of late and early NCR genes and identified some TFs that are co-expressed with early or late NCR genes. Although other methods like ChIP-Seq are needed to detect the direct interaction of particular TFs with NCR gene promoters, the TFs identified in our analysis seem relevant in light of the nature of the data. The TF WRKY40 regulates the expression of genes related to response to bacteria and refers to such GO biological processes as “defense response to bacteria” and as “defense response to fungus” (Chakraborty et al., 2018). Similarly, PTI5 is a TF regulating the expression of pathogen resistance genes (He et al., 2001; Gu et al., 2002). NAC969 is a negative transcriptional regulator of nodule senescence and regulates nodule premature senescence and abiotic stress tolerance in *M. truncatula*. Experiments using RNAi have shown that suppression of NAC969 expression leads to premature nodule senescence, which may be directly connected with the absence of early NCR gene expression (de Zélicourt et al., 2012).

The promoters of NCR genes in pea have putative binding sites for the TF NLP7, a member of the NIN-like protein family, which is accumulated in the nucleus in response to nitrate and directly regulates the production of CLE-RS2, a root-derived mobile peptide that acts as a negative regulator of nodule number (Nishida et al., 2018). The results obtained in our work suggest that NLP7 may also regulate the expression of “early” NCR genes. Other binding sites in promoters of NCR genes can be targets of auxin-response TF ARF2. It is known that ARF2 (together with ARF3 and ARF4) is involved in nodule organogenesis and rhizobia infection during nitrogen-fixing symbiosis in *M. truncatula*, regulating auxin-mediated developmental programs. Nallu et al. (2013) also identified ARF elements in the upstream region of NCR genes. Still, the question of whether ARF2 is the key regulator of NCR gene expression needs further investigation with the use of direct methods. Interestingly, promoters of genes that are co-expressed with NCR genes contain the same conservative motifs as promoters of NCR genes (Table 4). Since some of these co-expressed genes are related to hormonal signaling (nodulin-13 and gibberellin signaling DELLA protein LA) and autoregulation of nodulation (AON) (receptor-like protein CLAVATA2), this similarity of promoter regions may be the molecular genetic base of the possible link between long-distance signaling during nodulation and the expression of NCR genes.

The majority of NCR genes are expressed at 12 and 21 dpi, which coincides with the time of differentiation of free-living bacteria into bacteroids. However, a significant part of NCR genes has higher expression at 28 dpi and, especially, at 42 dpi (when the bacteroid differentiation is already completed). Obviously, the roles played by these late NCRs are different from those of early NCRs; for example, they could be involved in stricter control over the metabolic exchange between symbionts, or in the processes of senescence of symbiosomes. Interestingly, the spatial expression profiles indicate that the NCRs with pI 7.9–8.4 constitute the majority of NCR genes expressed in the early II zone (Figure 7B). A similar pattern was detected for NCR genes in *M. trunacatula* nodule microdissection as well (Montiel et al., 2017). Regarding the temporal expression profiles, at 28 dpi all groups of peptides (anionic, neutral, and cationic) are expressed at a high level, while for anionic peptides (pI < 6.5) two subgroups can be distinguished: those with (i) pI 4.4–5.4, with a maximal expression at 28 dpi and (ii) pI 5.4–5.9, with a maximal expression at 12 dpi. The specific roles of anionic NCR peptides pI 5.4–5.9 were at an early stage of nodule development and could be connected with its possible ability to neutralize the activity of cationic peptides for the precise tuning of TBD.

White nodules of SGEFix^–^-1 are characterized by abnormal morphological differentiation of bacteroids and premature degradation of nodule symbiotic structures (Tsyganov et al., 1998). Some of the “early” genes encoding NCR peptides whose expression in SGEFix^–^-1 does not differ from the wild type may be associated with the initiation of the terminal differentiation of bacteroids, while the “late” genes, whose expression is downregulated in SGEFix^–^-1, may be involved in completion of the TBD process, in nitrogen fixation, and in initiation (and/or suppression) of senescence of nodules.

The *sym33-3* allele is a weak allele that leads to the synthesis of truncated protein (Ovchinnikova et al., 2011). As a result, the SGEFix^–^-2 mutant is characterized by a leaky phenotype. In white nodules, most nodule cells are colonized cells containing infection threads without bacterial release. However, in some white nodules, bacterial release may occur and such cells become infected. Nonetheless, the bacteroids remain undifferentiated rod-shaped and gather in multibacteroid symbiosomes. This means that IPD3/CYCLOPS TF is a prerequisite for NCR peptide synthesis since its disruption leads to total downregulation of the expression of genes encoding NCR peptides in SGEFix^–^-2 nodules. Interestingly, the nodule phenotype of the *M. truncatula* mutant in the *ERN1* gene (Cerri et al., 2016) resembles, to an extent, the mutant phenotype of the SGEFix^–^-2 mutant. The co-expression and regulatory relationships identified between the *ERN1* gene and the genes encoding NCR peptides may indicate that *sym33-3* helps determine the expression of NCR genes through a change in *ERN1* gene expression.

The gene expression analysis demonstrated that in mutants, in comparison with wild-type nodules, the upregulated biological process terms comprised the defense response to bacteria and the regulation of defense reactions. Previously, an activation of strong defense responses in SGEFix^–^-1 and SGEFix^–^-2 mutant nodules was observed (Ivanova et al., 2015; Tsyganova et al., 2019). These responses included activation of defense response genes, suberinization, increased unesterified pectin deposition in infection threads walls, and enhanced cell wall material deposition around the vacuole. Further studies are needed to clarify the potential link between NCR gene expression and suppression of defense reactions in nodules.

A co-expression analysis using the data of wild-type and mutant nodule transcriptomes revealed several potential regulators of the NCR gene expression. However, the set of the identified TFs does not overlap with the set of TFs obtained on the data of gene expression in the wild-type SGE nodules at different time points. This can be explained by the fact that, in the wild-type nodules, the change in gene expression over time (from 12 to 28 dpi) is less pronounced as compared to the difference between wild-type and mutant nodules where the development of symbiotic structures is aborted. Possibly, transcriptomic studies involving other pea Fix^–^ mutants and younger wild-type nodules will contribute to identifying more potential regulators of NCR gene expression.

The number of known pea accessions in germplasm collections exceeds 70,000 (Smýkal et al., 2013). Apparently, further analysis of the NCR gene expression and polymorphism in a large set of pea accessions is needed to fully describe the role of this gene family in symbiosis, as single amino acid changes in some cultivars and genotypes may significantly change the physicochemical properties of NCR peptides and its effect on TBD and their viability in nodules.

## Conclusion

The gene family encoding NCR peptides in the pea, as in other IRLC legumes, is highly variable, and this variability leads to the production of a strong cocktail of defensin-like molecules in nodule cells. Although the antibiotic activity of a single NCR peptide may be minor, the toxicity of a peptide cocktail including molecules with different properties and modes of action against bacteria is much higher so that only compatible nodule bacteria could resist treatment with NCR peptides within nodules (Lima et al., 2020). The study of this antibiotic activity may help advance the formulation of new generations of antibiotics, an important effort in the light of increased pathogenic antibiotic-resistant bacteria. As it is known that NCR peptides penetrate bacterial cells and interfere with their vital functions, an antibiotic cocktail made of several NCR peptides may be formulated on the basis of calculated predictions related to the functions of relevant NCR peptides. Moreover, modification of the NCR gene and peptide sequences may result in stronger antibiotics, which may be useful in medicine and agricultural practices as well.

From the fundamental point of view, it is still not known whether mutations in NCR genes of pea might result in the Fix^–^ phenotype. Although no mutations in NCR genes in pea are known to date, the number of described and not sequenced genes still exceeds 10 (Tsyganov and Tsyganova, 2020). The mapping-based approach, which has recently demonstrated its feasibility (Zhernakov et al., 2019), may likely help identify such mutations in the near future and thus contribute to a more complete description of the NCR gene family in the pea.

## Data availability statement

The data presented in this study are deposited in the NCBI repository, accession numbers: PRJNA812957 and PRJNA853105.

## Author contributions

EZ, MK, IT, and VZ contributed to the conception and design of the study. EZ, MK, AA, MG, OK, and AZ searched for NCR genes, conducted polymorphism analysis, and computationally predicted properties of putative NCR peptides. EZ, MK, AA, EG, AS, AZ, OK, DR, and VZ carried out nodulation experiments and MACE RNAseq analysis. EZ and AA executed co-expression and promoter sequence analysis. PK and VT performed microdissection following RNAseq analysis. AT performed microscopy. VZ, EZ, and MK wrote the first draft of the manuscript. All authors contributed to the manuscript revision, read, and approved the submitted version.

## Footnotes

1
https://github.com/kjokkjok/NCRs_filter/

2
https://sourceforge.net/projects/bbmap/
