# Supplementary material for: A variable gene family encoding nodule-specific cysteine-rich peptides in pea (Pisum sativum L.)
Source: Front Plant Sci. 2022 Sep 14;13:884726. doi: 10.3389/fpls.2022.884726 (PMC9515463; doi:10.3389/fpls.2022.884726)
Supplement: Supplementary file 1 [file Data_Sheet_1.ZIP › Supplementary/Caption.docx]

**Supplementary Table 1****.** The list of putative NCR peptides of *P. sativum* with their sequences and basic physicochemical properties.

**Supplementary Figure 1.** Result of Principal Component Analysis of RNA sequencing data from nodules.

**Supplementary Figure 2.** Heatmap showing pairwise similarity of NCR peptide sequences in P. sativum

**Supplementary Figure 3.** Distribution of SNPs by gene regions in SGE and Caméor genotypes in comparison to Frisson.

**Supplementary Figure 4.** Co-expression analysis of DEGs and their Gene Ontology enrichment analysis in SGEFix^--^-1 and SGEFix^--^-2 mutant
