# Supplementary material for: A variable gene family encoding nodule-specific cysteine-rich peptides in pea (Pisum sativum L.)
Source: Front Plant Sci. 2022 Sep 14;13:884726. doi: 10.3389/fpls.2022.884726 (PMC9515463; doi:10.3389/fpls.2022.884726)
Supplement: Supplementary file 1 [file Data_Sheet_1.ZIP › Supplementary/Supplementary_Figure2.pdf]

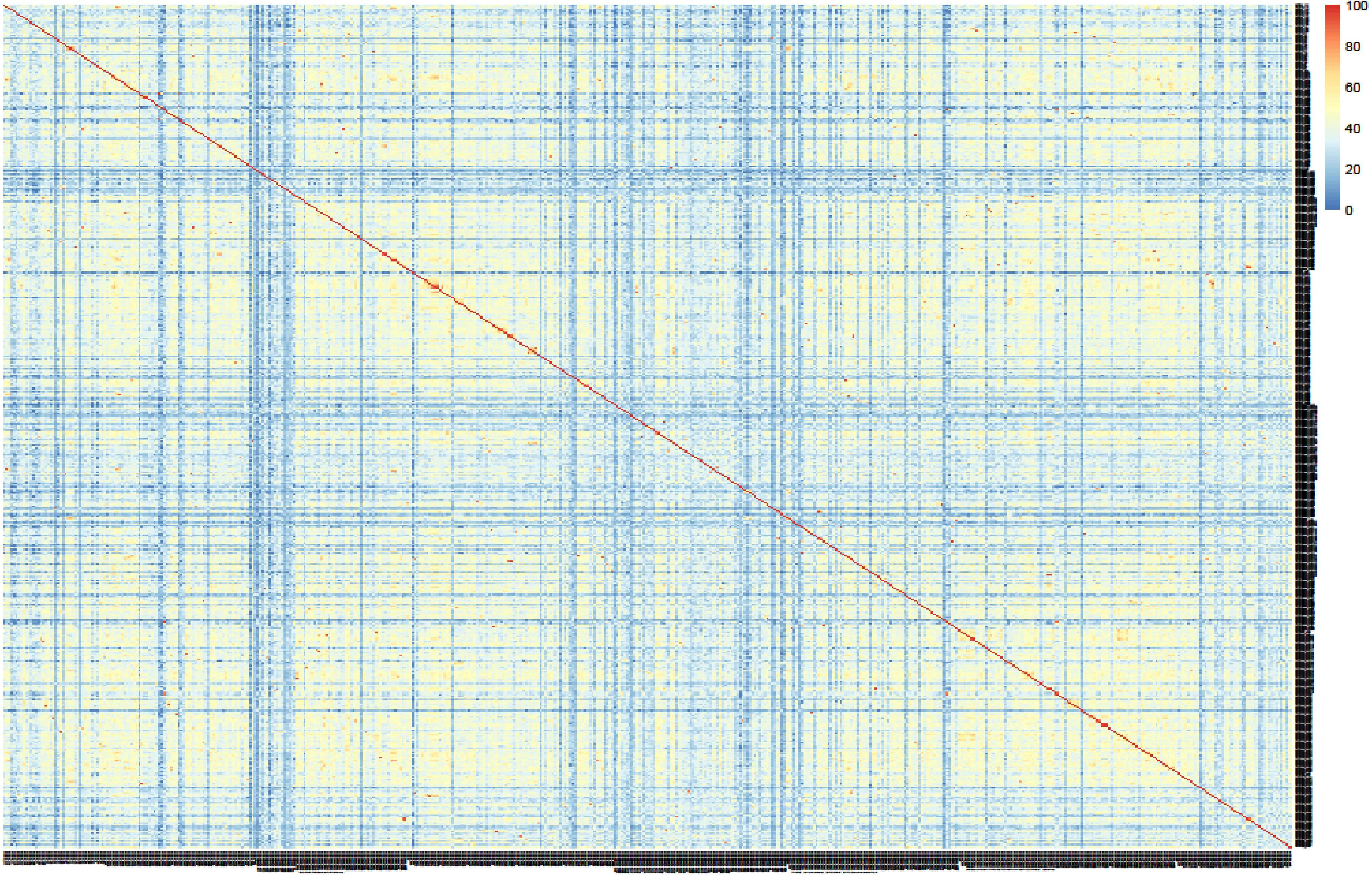

Supplementary Figure 2. heatmap showing pairwise similarity of NCR peptide sequences in *P. sativum*
