# Supplementary material for: A variable gene family encoding nodule-specific cysteine-rich peptides in pea (Pisum sativum L.)
Source: Front Plant Sci. 2022 Sep 14;13:884726. doi: 10.3389/fpls.2022.884726 (PMC9515463; doi:10.3389/fpls.2022.884726)
Supplement: Supplementary file 1 [file Data_Sheet_1.ZIP › Supplementary/Supplementary_Figure4.pdf]

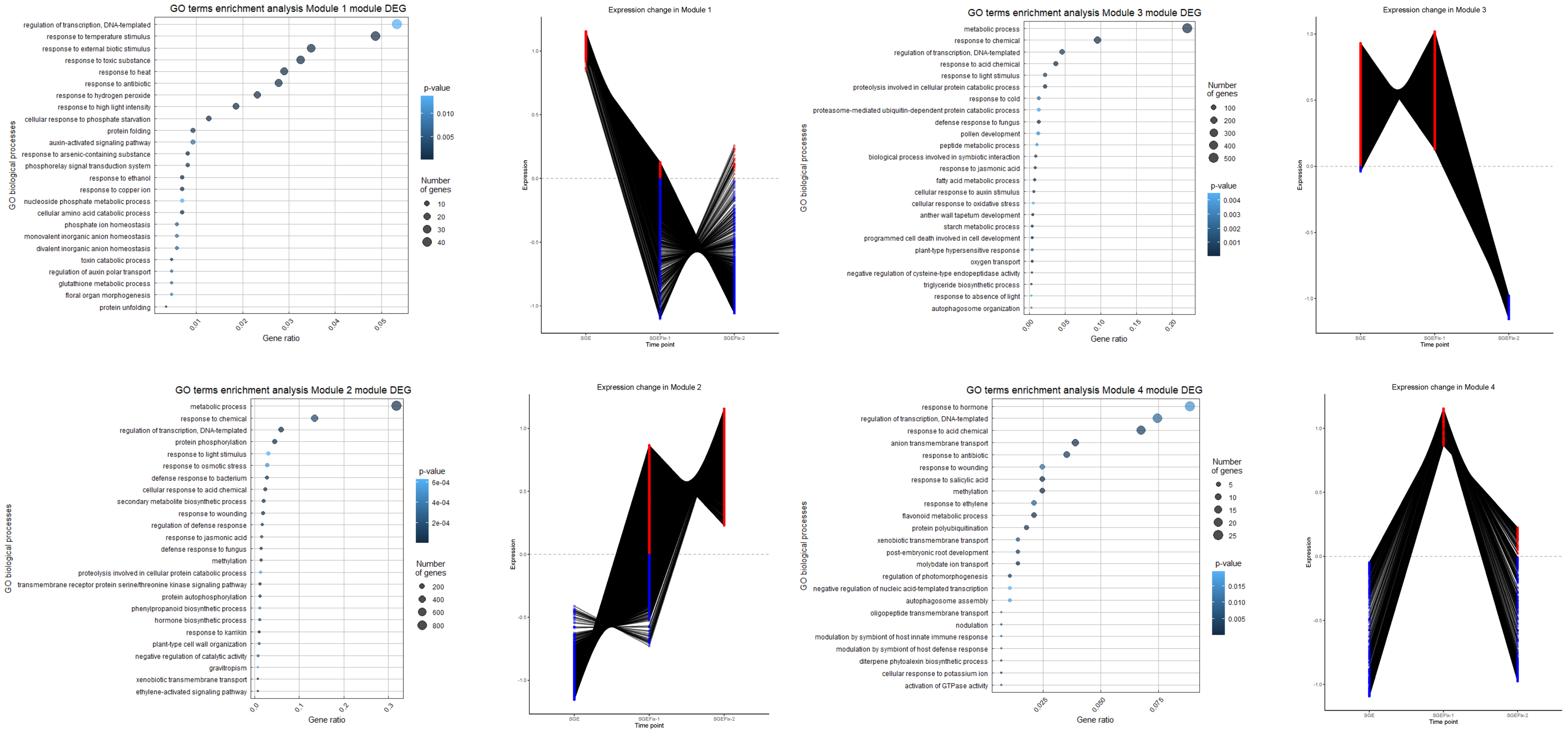

Supplementary Figure 4. Co-expression analysis of DEGs and their Gene Ontology enrichment analysis in SGEFix^{--}-1 and SGEFix^{--}-2 mutants
